# Emergent Criticality in Coupled Boolean Networks

**DOI:** 10.3390/e25020235

**Published:** 2023-01-27

**Authors:** Chris Kang, Madelynn McElroy, Nikolaos K. Voulgarakis

**Affiliations:** 1 Department of Mathematics and Statistics, Washington State University, Pullman, WA 99164, USA; 2 Voiland School of Chemical Engineering and Bioengineering, Washington State University, Pullman, WA 99164, USA

**Keywords:** coupled Boolean networks, multilayer Ising model, cell differentiation, symmetry breaking, self-tuned criticality

## Abstract

Early embryonic development involves forming all specialized cells from a fluid-like mass of identical stem cells. The differentiation process consists of a series of symmetry-breaking events, starting from a high-symmetry state (stem cells) to a low-symmetry state (specialized cells). This scenario closely resembles phase transitions in statistical mechanics. To theoretically study this hypothesis, we model embryonic stem cell (ESC) populations through a coupled Boolean network (BN) model. The interaction is applied using a multilayer Ising model that considers paracrine and autocrine signaling, along with external interventions. It is demonstrated that cell-to-cell variability can be interpreted as a mixture of steady-state probability distributions. Simulations have revealed that such models can undergo a series of first- and second-order phase transitions as a function of the system parameters that describe gene expression noise and interaction strengths. These phase transitions result in spontaneous symmetry-breaking events that generate new types of cells characterized by various steady-state distributions. Coupled BNs have also been shown to self-organize in states that allow spontaneous cell differentiation.

## 1. Introduction

The development of multicellular organisms depends largely on the ability of stem cells to self-replicate indefinitely (proliferation) and differentiate into specialized cells (pluripotency). Macroscopically, the outcome of this process is predictable in an almost deterministic fashion, i.e., they are almost always primed to functional cells in their lineage. Microscopically, stem cell dynamics appear to be stochastic due to the molecular nature of gene expression. The intrinsic noise causes noticeable cell-to-cell variability in stem cell populations [1]. Although the functional role of gene expression noise and cell variability remains elusive, many have suggested that gene expression noise and cell variability are integral to stem cell pluripotency. Huang et al. proposed that pluripotent (multipotent) stem cells are a “balanced, undecided state” of multiple gene expression patterns [2,3]. Noise and environmental signaling simply destabilize this state and force stem cells to overexpress only in certain genes, thus acquiring a new type of cell with specialized functions. Others have argued that this transition may only emerge or, at least, is significantly amplified in stem cell populations due to the interplay between cell–cell interaction, gene expression noise, and environmental signals [4,5,6]. Irrespective of the detailed mechanism, the general theory states that pluripotency is a state of higher symmetry (i.e., a higher amount of coexpressed genes), while differentiation leads to states of lower symmetry (lower amount of similarly expressed genes).

Symmetry breaking is the hallmark of disorder–order phase transitions in large physical systems of interacting agents [7]. The system transitions from a state of higher symmetry (disorder) to a state of lower symmetry (order) by reducing the intrinsic noise (control parameter) below a critical point. Examples of this type of phase transition include paramagnetic–ferromagnetic or liquid–solid phase transitions. In general, the symmetry breaks spontaneously at the critical value of a control parameter. Below the critical value, the system becomes non-ergodic, and the ordered state of the system is perfectly stabilized. The parameter that measures the order of the system (order parameter) may change continuously (second order) or discontinuously (first order) [7,8,9]. At the critical point, and depending on the order of the phase transition, the system possesses intriguing dynamic properties, such as strong spatiotemporal correlations, maximum susceptibility to external stimuli, optimal information transfer, robustness to random perturbations, coexistence of phases, memory effects, etc. It is important to note that the results mentioned above are applicable only within the thermodynamic limit (infinitely large systems). There is no strong ergodicity breaking for small systems, and, as a consequence, the system may fluctuate between different states. This fluctuation in states might also be a theoretical interpretation of the important cell-to-cell variability observed in small stem cell colonies. The essential hypothesis put forward is [10] could the stem cells possibly embrace and properly buffer the intrinsic noise to drive the system through its critical point?

Here, we attempt to address this question theoretically in terms of standard statistical mechanics [4,5,11]. Every cell in the population is modeled by isogenic Boolean networks (BNs), while the cell–cell interaction is governed by a multilayer Ising type of Hamiltonian that accounts for paracrine and autocrine signaling, as well as external interventions. The system’s evolution is given by a hybrid Monte Carlo (MC) method and synchronous BN dynamics. We illustrate that time-dependent mixtures of steady-state distributions can effectively represent the collective dynamics of BNs. This problem can be solved numerically by implementing standard optimization approaches, spectral decomposition methods, or unsupervised machine learning algorithms based on non-negative matrix factorization (NNMF). Statistical analysis reveals that coupled BNs exhibit signatures of first- and second-order phase transition. These transitions are accompanied by spontaneous symmetry-breaking events that can be interpreted as cell differentiation. Due to strong fluctuations in the cellular state close to the critical points, the system may exhibit significant heterogeneity [12]. Coupled BNs can efficiently tune themselves to a critical state of spontaneous symmetry breaking through a feedback differential equation that modulates the system’s intrinsic noise.

This paper is organized as follows. Section 2 describes the model and the methods applied in this work. Specifically, Section 2.1 briefly presents the Boolean network approach and introduces the concept of a dynamic control kernel. Section 2.3 introduces the multilayer Ising model and discusses the simulation approach. The various methods used to detect different types of cells are presented in Section 2.4. Section 3 is devoted to numerical analysis and the proposed equation for a self-tuning mechanism. Finally, Section 4 concludes the findings and discusses future directions.

## 2. Models and Methods

### 2.1. Boolean Networks

In this work, we model the gene regulations of a cell as a Boolean network (BN) subject to intrinsic noise. BNs consider each molecular regulator as a binary node that can be “on” (active) or “off” (inactive). Each node of the network regulates neighboring nodes through predefined deterministic rules. As a finite deterministic system, BNs can have different attractors, commonly interpreted as possible functions of the cell. However, introducing random perturbations that mimic gene expression noise makes the dynamics ergodic, and all BNs have their own steady-state distribution peaked around the attractors [13]. Such distributions can be used to explore the complexity of BN dynamics. Depending on the statistics of the predefined deterministic rules, BNs can exhibit order, critical, or chaotic dynamics. Despite its simplistic nature, BNs have been used to effectively model various experimental observations, such as the yeast transcriptional network [14,15], the wild-type gene expression patterns (segment-polarity) of Drosophila melanogaster [16], the signaling system within capillary endothelial cells [17], and the T-cell signaling pathway [18].

A Boolean network is defined on a set of *n* interacting nodes N={1,…,n} representing regulatory genes. Each node *i* is assigned a Boolean value $x_{i}\in \left\{0,1\right\}$ (inactive and active, respectively). Each gene *i* is regulated by $k_{i}\leq n$ genes $J_{i}=\left\{j_{1},\ldots ,j_{k_{i}}\right\}\subseteq N$. A synchronous update of the state vector $x\left(t\right)={\left\{x_{i}\left(t\right)\right\}}_{i\in N}$ at discrete time *t* is given by

(1) $$\begin{array}{c} x\left(t\right)={\left\{f_{i}\left(y_{i}\left(t-1\right)\right)\right\}}_{i\in N}, \end{array}$$

where $y_{i}\left(t-1\right)={\left\{x_{j}\left(t-1\right)\right\}}_{j\in J_{i}}$ is the state vector of the interacting nodes at the previous time and $f_{i}:{\left\{0,1\right\}}^{k_{i}}\to \left\{0,1\right\}$ is the Boolean function. At any given time *t*, the state of BN is defined by the state vector x(t) or the decimal-encoded state, s(t)∈S, of x(t). Here, $S=\{1,\ldots ,2^{n}\}$ is the decimal representation of the states indexed from 1 to $N=2^{n}$ possible configurations of the state space.

In reality, it is uncommon to have full knowledge of the specific gene connectivity and Boolean functions of a GRN. For this purpose, Kaufmann proposed an *ensemble approach* to study GRNs, where statistical averages of random Boolean networks (RBNs) are taken. In an RBN, for all i∈N (a), the number of connections is the same (that is, $k_{i}=k$), (b) the interacting nodes are randomly chosen, and (c) for any input $y_{i}$, $f_{i}\left(y_{i}\right)=1$ with probability bias of *p* and $f_{i}\left(y_{i}\right)=0$, otherwise. By varying the control parameters *k* and *p*, RBNs undergo a phase transition between order and chaotic network dynamics. The *edge of chaos* exists at the critical connectivity $k_{c}=1/\left[2p(1-p)\right]$ [19,20].

As the dynamics of a BN are deterministic in finite space, the network eventually entails repetition of gene states. These *dynamical attractors* are an important feature of complex systems and have long been established as gene expression patterns that characterize cell types [21,22,23]. One way to observe attractors of networks with a reasonably small number of genes is to numerically approximate the steady-state distribution through model simulations [24,25,26]. In a BN, Monte Carlo simulations can approximate the steady-state probability distribution of gene states by introducing a random perturbation to the synchronous update of the network [13]. A possible BN realization with perturbation is to update gene states according to Equation (1) with probability $1-{\left(1-q\right)}^{n}$ and update with $x_{i}\left(t\right)=\gamma_{i}$, otherwise, where i∈N, $\gamma_{i}\in \left\{0,1\right\}$ and $q=P\{\gamma_{i}=1\}$. We note that the original formulation of random perturbation in [13] is a binary “flip” of a randomly selected gene. Here, we perturb by updating the entire gene set x with a random vector of binary values $\left\{\gamma_{1},\ldots ,\gamma_{n}\right\}$. With perturbation, there is a nonzero probability of arriving at any state, and thus the Boolean network is an ergodic Markov chain where the states converge given sufficient time [27]. We note that, although random perturbation is used here to require ergodicity of the dynamics, it also mimics the gene expression noise observed in biological systems. For example, in *Escherichia coli*, the gene expression noise modeled as stochastic dynamics has been well studied to be necessary for its regulation, fluctuation of transcription rate, and cell division [28,29,30,31].

**Figure 1:**
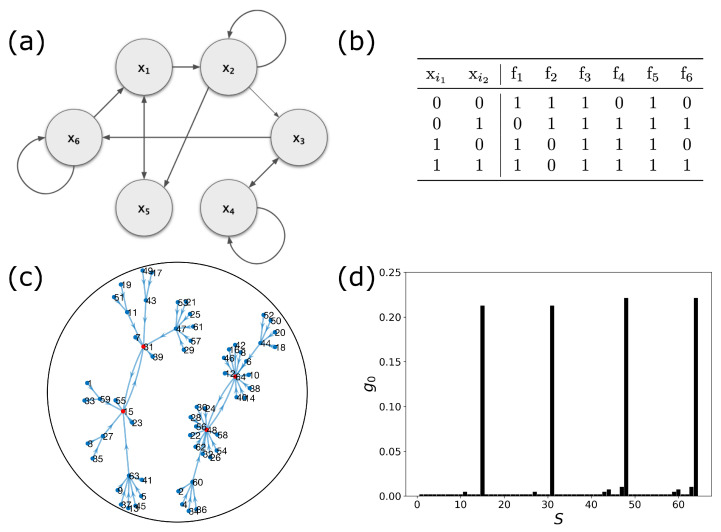
A 6-gene network with k=2, and the bias of p=0.5. (**a**) Wiring diagram of the network. (**b**) Truth table of Boolean functions. (**c**) State transition diagram. (**d**) Steady-state distribution, $g_{0}\left(s\right)$, with perturbation q=0.1.

Figure 1 shows the wiring diagram, truth table, state transition diagram, and steady-state distribution of a 6-gene Boolean network with connectivity of k=2 and bias p=0.5 with the perturbation probability of q=0.1. Throughout this work, we use this model 6-gene network for demonstration.

### 2.2. Dynamic Control Kernel

The control of gene expression is a highly desirable action in many areas of molecular biology. For example, targeted drug delivery or the knockdown effect in gene therapy requires persistent external influence on a cell to achieve desired gene expression. In the context of BNs, there are numerous different control strategies. Shmulevich et al. initially proposed the optimal *intervention* strategy of probabilistic Boolean networks by altering Boolean functions [13]. For this type of intervention, one is often interested in finding the best candidate genes to intervene by minimizing the mean first-passage time. However, in recent work, direct modification of gene states to drive the dynamics to the desired attractors has been proposed [32]. Here, we focus on a specific notion of control, *pinning*, also known as node-state override, where the gene state is permanently fixed. Pinning differs from the original formulation of intervention in that Boolean functions are preserved for all genes except those that are kept static. This concept was first introduced by Serra et al. and was described as the *knock-out* of genes with an avalanche effect [33]. Kim et al. formalized the *control kernel* (CK) of a network as the minimal set of genes whose pinning reshapes the dynamics such that the basin of the attractor becomes the entire configuration space [34]. In a pinned network, only a small fraction of the total number of network nodes depends on the topological and logical characteristics of the network. Experimentally, Joo et al. have explored the control of a single gene as an external input for a 5-gene network that captures the molecular mechanisms and the cell-state transition from an epithelial to a mesenchymal stem cell [35].

Let r≤n be the cardinality of a CK set. Without loss of generality, we can assume that the CK nodes are the first *r* nodes of a BN. We define the state vector of the CK and the remainder network as $x_{c}=\left\{x_{1},\ldots ,x_{r}\right\}$ and $x_{o}=x-x_{c}$, respectively. The set of decimal-encoded $2^{r}$ states of CK is $W_{r}=\left\{1,\ldots ,2^{r}\right\}$. We denote the ordered sets of the pinned values of CK as $X_{w}$, where $w\in W_{r}$. For example, $X_{1}=\left\{x_{1}=0,x_{2}=0,\ldots ,x_{r-1}=0,x_{r}=0\right\}$, $X_{2}=\left\{x_{1}=0,x_{2}=0,\ldots ,x_{r-1}=0,x_{r}=1\right\}$, etc. Then, the dynamics of an RBN are as follows:

(2) $$\begin{array}{c} \left\{\begin{array}{cc} x_{c}\left(t\right) & =X_{w}\\ x_{o}\left(t\right) & ={\left\{f_{i}\left(y_{i}\left(t-1\right)\right)\right\}}_{i>r}. \end{array}\right. \end{array}$$

We reserve the index w=0 to describe the unpinned dynamics, that is, $X_{0}=\{f_{i}{\left(y_{i}\left(t-1\right)\right\}}_{i\leq r}$. In other words, when w=0, Equation (3) is Equation (1).

**Figure 2:**
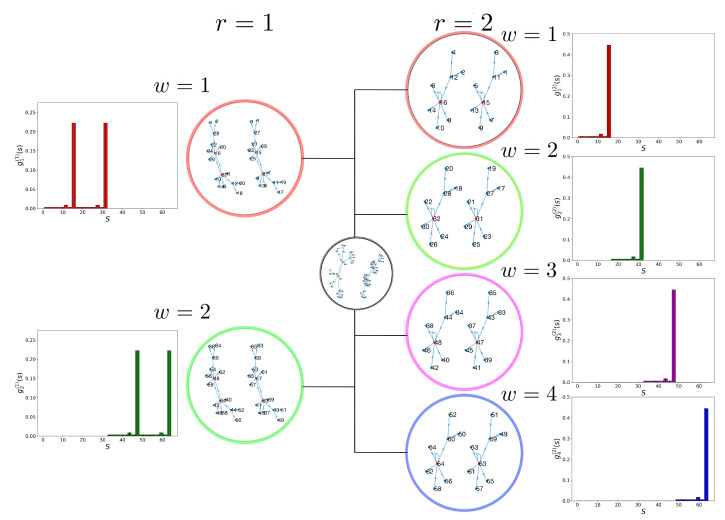
(**Left**) Pinned BN with r=1; $x_{1}$ is either 0 or 1. The two resulting steady-state distributions $g_{1}^{\left(1\right)}\left(s\right)$ and $g_{2}^{\left(1\right)}\left(s\right)$ have partitioned state spaces and preserve attractor points. (**Right**) Pinned BN with r=2; $x_{1}$ and $x_{2}$ are pinned to either 0 or 1. The four resulting steady-state distributions $g_{1}^{\left(2\right)}\left(s\right)$, $g_{2}^{\left(2\right)}\left(s\right)$, $g_{3}^{\left(2\right)}\left(s\right)$, and $g_{4}^{\left(2\right)}\left(s\right)$, once again, have partitioned state spaces and preserve attractor points.

For each $w\in W_{r}$, the pinning procedure generates a new steady-state distribution $g_{w}^{\left(r\right)}={\left\{g_{w}^{\left(r\right)}\left(s\right)\right\}}_{s\in S}$ that is attributed to a new type of cell. Thus, each CK set can generate up to $2^{r}$ new types of cells. An interesting property of this control approach is that it naturally partitions the state space, S, into $2^{r}$ equivalent sets. As a consequence, for i≠j, we have the orthogonal condition

(3) $$\begin{array}{c} \langle g_{i}^{\left(r\right)}\;,g_{j}^{\left(r\right)}\rangle =0, \end{array}$$

where $\langle g_{i}^{\left(r\right)}\;,g_{j}^{\left(r\right)}\rangle =\sum_{s\in S}g_{i}^{\left(r\right)}\left(s\right)g_{j}^{\left(r\right)}\left(s\right)$ is the scalar product of $g_{i}^{\left(r\right)}$ and $g_{j}^{\left(r\right)}$. This property is illustrated in Figure 2, where we applied the pining procedure to the 6-gene model BN. In the case of r=1, pinning the CK to either $X_{1}$ or $X_{2}$ generates two nonoverlapping steady-state distributions $g_{1}^{\left(1\right)}\left(s\right)$ and $g_{2}^{\left(1\right)}\left(s\right)$, respectively (Figure 2 (left)). Similarly, a CK of r=2 generates four disjoint steady-state distributions $g_{1}^{\left(2\right)}\left(s\right)$, $g_{2}^{\left(2\right)}\left(s\right)$, $g_{3}^{\left(2\right)}\left(s\right)$, and $g_{4}^{\left(2\right)}\left(s\right)$ (Figure 2 (right)).

Here, we define dynamic CK as $x_{c}\left(t\right)=X_{\eta_{t}}$, where $\eta_{t}$ is a discrete-time deterministic or stochastic process with state space $W\subseteq \{\left\{0\right\},W_{r}\}$. Let g(s) be the new steady-state distribution as a result of $X_{\eta_{t}}$. If the mean waiting time in a given state of $\eta_{t}$ is finite and long enough, the distribution of the cell states will be a mixture of all $g_{w}$s, i.e.,

(4) $$g\left(s\right)=c_{0}g_{0}\left(s\right)+\sum_{w\in W_{r}}c_{w}^{\left(r\right)}g_{w}^{\left(r\right)}\left(s\right),$$

where $c_{w}$s are the scalar weights of the respective $g_{w}$s.

**Figure 3:**
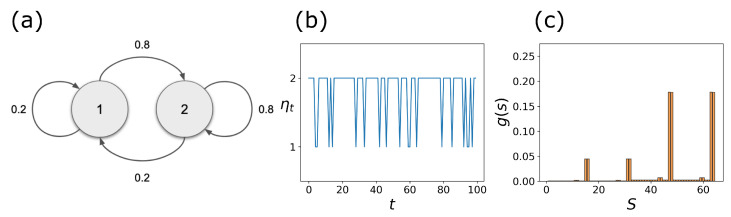
Stochastic pinning of the BN: (**a**) transition diagram for a two-state, $W_{1}=\left\{1,2\right\}$, stochastic pinning process; (**b**) first 100 sampled values of the stochastic process $\eta_{t}$; and (**c**) steady-state distribution of the stochastic CK, g(s), obtained after $T=10^{5}$ simulation steps.

To illustrate this point, we set r=1 and assume that $\eta_{t}\in W_{1}=\left\{1,2\right\}$ is a discrete-time Markov chain process with the transition diagram shown in Figure 3a. Figure 3b shows the time evolution of $\eta_{t}$ for the first 100 time steps. It is clear that $\eta_{t}=2$ is sampled more than $\eta_{t}=1$. The resulting steady-state distribution g(s), shown in Figure 3c, is a *mixture* of $g_{1}^{\left(1\right)}\left(s\right)$ and $g_{2}^{\left(1\right)}\left(s\right)$ with the corresponding weights $c_{2}>c_{1}$. Here, we interpret this as that *the new cell type g(s) resulting from stochastic pinning is a weighted mixture of cell types $g_{1}^{\left(1\right)}$ and $g_{2}^{\left(1\right)}$*. This is true as long as the transition rates are not “too fast.” In Section 2.4, we take advantage of the disjoint property of the $g_{w}$s to develop a spectral decomposition method that provides these weights. Other methods, such as linear optimization and non-negative matrix factorization (NNMF), are discussed in the case where $g_{1}^{\left(1\right)}\left(s\right)$ and $g_{2}^{\left(1\right)}\left(s\right)$ are no longer disjoint under different CK rules.

### 2.3. Coupled Boolean Networks

The study of coupled BNs has gained increased interest in recent decades. Villani et al. and Serra et al. have shown that increasing interactions between RBNs results in more disordered states [36,37]. Damiani et al. revealed that short-distance interacting RBNs display robust generic properties [38]. The collective behaviors of coupled BNs have been investigated in [26,39,40]. In particular, [39] showed that the pattern formation in the tissues of BNs is the most information-rich in the near-critical complexity domain, while in [26], the authors showed that the pattern formation of long-term steady states is most often observed in networks of critical dynamics. In the most recent study by Kim et al., *multilayer* RBNs were investigated, where isogenic GRN were coupled according to a random selection of topology in silico with activation rules from [39]. They showed that a multilayer RBN structure facilitated the production of antifragile systems [40].

In this work, we model stem cell populations as *L*-coupled isogenic BNs (tissue). Let $x_{i,m}$ represent the *m*-th gene of the *i*-th cell. Each cell *i* is allowed to interact with a set of other cells denoted by $\Gamma_{i}$. For each Boolean variable σ∈{0,1}, we define a linear transformation $\bar{\sigma }=2\sigma -1\in \left\{-1,1\right\}$. We assume that BNs interact with each other through their CK according to the multilayer Ising Hamiltonian:

(5) $$H_{r}=-\sum_{m=1}^{r}\sum_{i=1}^{L}\left[\sum_{j\in \Gamma_{i}}J_{ij}{\bar{x}}_{i,m}{\bar{x}}_{j,m}+h_{0}{\bar{x}}_{i,m}{\bar{f}}_{i,m}+h{\bar{x}}_{i,m}{\bar{\psi }}_{m}\right].$$

In terms of a standard spin model, the first term of Equation (5) is the interaction between cells *i* and *j*, where $J_{ij}$ is the strength of the interaction. It is simply the Hamiltonian of *r*-independent Ising models. The second and third terms represent local external fields acting on the control kernel, where *h* and $h_{0}$ are the corresponding strengths. In our coupled BN model, the first term describes paracrine signaling with neighboring cells. The second term describes the cell’s tendency to follow the dynamics of its original cell (${\bar{f}}_{i,m}$). By default, it is time-dependent and indirectly couples the *r* Ising models. The third may describe autocrine signaling and/or external interventions (${\bar{\psi }}_{m}$) and can be static or time-dependent.

Cell–cell communication or cell interaction with external signals is facilitated by diffusing signaling molecules. To capture this stochastic process, we introduce an additional type of intrinsic noise, denoted as *T*, which acts at the population level. The analog of *T* in standard statistical mechanics is the temperature of the system.

To simulate the system, we use the standard Metropolis algorithm for the coupled CKs and the Equation (2) for the synchronous BN update. During an MC step, the $N_{MC}\leq L$ subset of nodes, $x_{i,m}$, for each 1≤m≤r, is chosen randomly. We sequentially flip their state. The new state is accepted with probability min(1,exp(−ΔE/T), where ΔE is the energy difference between the current and the attempted state. After each MC step, all BN states $x_{o}$ are updated.

### 2.4. Detecting Cell Types

In Section 2.2, we illustrated that *dynamic* CKs can generate a new steady-state distribution as a mixture of the distributions. Let the vector G(t) be the instantaneous distribution of the states of all cells in a population and

(6) $$\tilde{G}\left(t\right)=c_{0}\left(t\right)g_{0}+\sum_{w\in W_{r}}c_{w}^{\left(r\right)}\left(t\right)g_{w}^{\left(r\right)},$$

be the approximation given by Equation (4). We define the relative error as $\epsilon \left(t\right)=||G\left(t\right)-\tilde{G}\left(t\right)\left|\right|/N$, where ||.|| denotes the standard Euclidean norm. Finding the coefficients $c_{0}$ and $c_{w}^{\left(r\right)}$ is a linear optimization problem with two constraints: (a) $0\leq c_{0}\leq 1$ and $0\leq c_{w}^{\left(r\right)}\leq 1$ for all *w*, and (b) $c_{0}+\sum_{w}c_{w}^{\left(r\right)}=1$. These two constraints allow us to interpret the coefficients as the number density or fraction of each cell type in the population.

In the case $h_{0}=0$, the population dynamics are dictated by the network of interacting CKs and external intervention. Thus, all CKs are expected to be in a state $w\in W_{r}$. In other words, $c_{0}=0$. This assumption can significantly simplify the computation of coefficients. Taking advantage of the nonoverlapping (disjoint) property of $g_{w}$, we define a set of orthonormal vectors as ${\hat{g}}_{w}^{\left(r\right)}=g_{w}^{\left(r\right)}/\langle g_{w}^{\left(r\right)}\;,g_{w}^{\left(r\right)}\rangle$. Then, one can use a simple spectral decomposition method to obtain the coefficients:

(7) $$c_{w}^{\left(r\right)}\left(t\right)=\langle G\left(t\right)\;,\;{\hat{g}}_{w}^{\left(r\right)}\rangle .$$

If the error ϵ(t) is not sufficiently small, the assumption of Equation (6) breaks down. In such cases, machine learning algorithms can be implemented to decompose G and gain insight into the dynamics of the system. Here, since the elements of G are non-negative, we use non-negative matrix factorization (NNMF) [41]. In short, NNMF can decompose a non-negative matrix $G_{N\times T}$ into a product of two non-negative matrices $\Omega_{N\times K}$ and $E_{K\times T}$. Here, T is the simulation time, and *K* is the rank of the decomposition to be determined. The matrix $\Omega_{N\times K}$ provides K-different steady-state distributions, which can also be interpreted as new types of cells. The corresponding time-dependent density numbers are stored in $E_{K\times T}$.

## 3. Results

This section presents the results of our numerical simulations based on the hybrid Monte Carlo and synchronous BN update method. We assume that we have a simple 2D tissue with periodic boundary conditions. The set $\Gamma_{i}$ consists of the four nearest neighbors of the *i*th cell. For simplicity, we choose $J_{ij}=J\geq 0$.

Some interesting limits of the multilayer Ising model include:

**Case 1**. If $h_{0}=0$, the CK is not affected by unpinned dynamics. Thus, the Hamiltonian reduces to the standard *r*-layer Ising model under the influence of an external field.
**Case 2**. If J=0 and h=0, then Equation (5) describes *L*-uncoupled BNs (non-interacting cells).
**Case 3**. For $h_{0}\approx J$, the *r* Ising layers are coupled through the dynamics of each BN. This is a nontrivial, bidirectional, and time-dependent nonlinear coupling.

In this work, we set J=1 and neglect the third term in Equation (5). Thus, the control parameters are intrinsic noise (“temperature”) *T* and local coupling $h_{0}$. For each set of parameters, we first equilibrate the system for $t_{eq}$ time steps and then compute the time average of an observable A(t) as $A=\sum_{t=1}^{T}A\left(t\right)/T$, where T is the sampling time. The time average of the numerical error ϵ is always less than $10^{-2}$. In Section 3.1, we use the linear optimization technique to compute the coefficients of Equation (6), while in Section 3.2, where we assume that $h_{0}=0$, we implement the spectral decomposition method from Section 2.4.

In what follows, we present the results for r=1 or r=2. Unless otherwise stated, $T=t_{\mathrm{eq}}=10^{4}$. The parameters values are summarized in Appendix A.

### 3.1. Spontaneous Cell Differentiation

This section examines the behavior of the system as a function of the control parameters *T* and $h_{0}$. Specifically, we show that varying the control parameters causes the system to undergo a series of spontaneous symmetry-breaking events. It should be noted that many biological phenomena, such as the stem cell differentiation process, are precisely explained by this phase transition. For example, the first step in the core embryonic stem cell cycle is the organization of the pluripotent state in cells [42]. Upon dissolution of pluripotency, stem cells reach a critical state, at which point they undergo symmetry breaking. The stem cell population spontaneously (and collectively) undergoes a cell fate specification for differentiated specialized cells.

**Figure 4:**
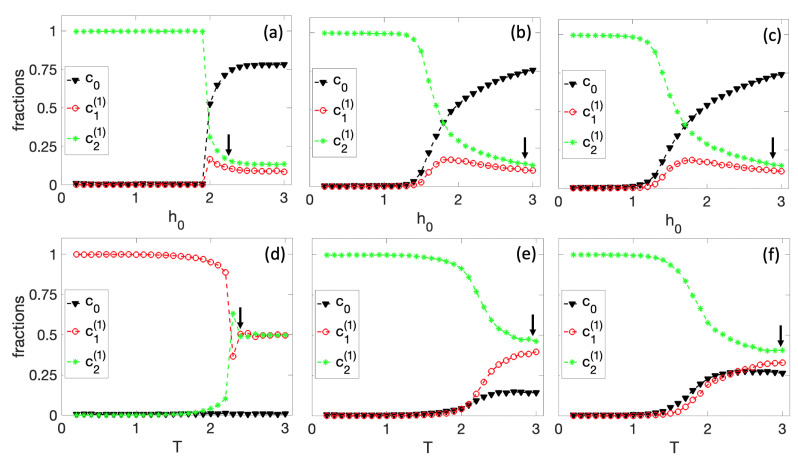
Differentiation process for r=1 and a 50×50 tissue size. First row: spontaneous differentiation process as a function of $h_{0}$ for (**a**) T=0.25, (**b**) T=1, and (**c**) T=1.25. Second row: spontaneous differentiation process as a function of *T* for (**d**) $h_{0}=0$, (**e**) $h_{0}=0.5$, and (**f**) $h_{0}=1$. The arrow approximately indicates the differentiation starting point.

In the first set of simulations, shown in Figure 4, we fix r=1. In Figure 4a–c, we kept the temperature constant at T=0.25, T=1, and T=1.25, respectively, and varied the parameter $h_{0}$. Specifically, we started with a high value of $h_{0}=3$, where we expected the fraction of the original cell $c_{0}$ to be dominant and gradually reduced it to $h_{0}=0$. For $h_{0}=3$, the initial conditions of all nodes in the tissue were randomly chosen. The final state of this simulation is the initial condition for the next value of the local field $h_{0}$, and so on. For high values of $h_{0}$, we see that $c_{0}$ is the most dominant fraction in the colony. As $h_{0}$ decreases, $c_{0}$ drops to a critical value where spontaneous differentiation occurs. An arrow in Figure 4 approximately indicates the starting point of the differentiation process. Note that the system always differentiates to the $g_{2}^{\left(1\right)}$ cell. This decision is related to the structure of the original BN. As seen in Figure 1d, the steady-state distribution is slightly higher in the second half of the state space, and this small difference always favors $g_{2}^{\left(1\right)}$. However, different BNs may exhibit alternative phenotypic behaviors. This transition is smooth for high temperatures (T=1 and T=1.25). However, the differentiation seems rather steep for low temperatures (T=0.5).

In Figure 4d–f, we fixed the local field constant at $h_{0}=0$, $h_{0}=0.5$, and $h_{0}=1$, respectively, and simulated with various temperature points from a high value of T=3 to a low value of T=0.1. Here, we also observe a differentiation at a critical temperature indicated by the arrow, which consistently rules in favor of $g_{2}^{\left(1\right)}$ for nonzero $h_{0}$. However, for $h_{0}=0$ (Figure 4d), the original cell has no influence on the dynamics of the system, and the cells can differentiate to $g_{1}^{\left(1\right)}$ or $g_{2}^{\left(1\right)}$ with an equal probability. In Figure 4e, we show an example where the cell differentiates into $g_{1}^{\left(1\right)}$. As a matter of fact, in this limit, Equation (5) is the standard Ising model. If one defines the order parameter 〈M〉, where *M* is the time average of $\left|M_{t}\right|=\left|c_{2}^{\left(1\right)}\left(t\right)-c_{1}^{\left(1\right)}\left(t\right)\right|$, and the brackets indicate the ensemble average, it can be shown that the system belongs to the same universality class as the 2D Ising model.

**Figure 5:**
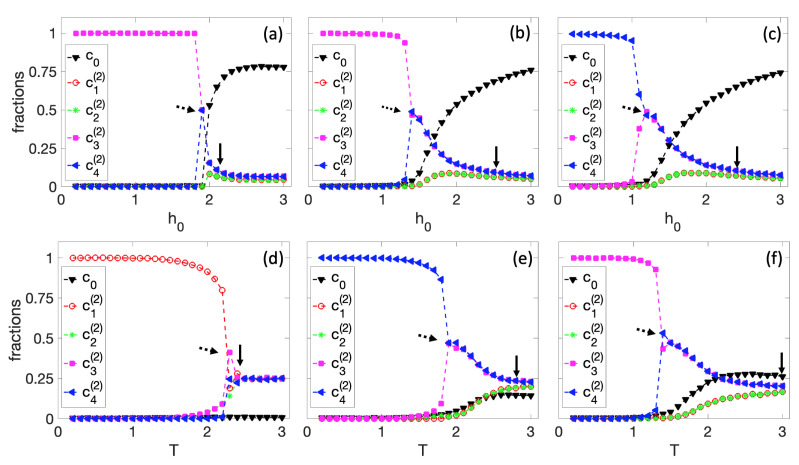
Differentiation process for r=2 and on a 50×50 tissue. First row: spontaneous differentiation process as a function of $h_{0}$ for (**a**) T=0.25, (**b**) T=1, and (**c**) T=1.25. Second row: spontaneous differentiation process as a function of *T* for (**d**) $h_{0}=0$, (**e**) $h_{0}=0.5$, and (**f**) $h_{0}=1$. The arrow and dashed arrow denote approximate starting points of two different differentiation processes.

In Figure 5, we performed the same simulations as in Figure 4 but for r=2. In this case, the BN has four potential steady states to differentiate. As observed in all the graphs in Figure 5, two consecutive cell differentiations exist; the arrow indicates the first sequence of differentiation, and the dashed arrow indicates the second. First, there is a differentiation between $\left\{g_{1}^{\left(2\right)},g_{2}^{\left(2\right)}\right\}$ and $\left\{g_{3}^{\left(2\right)},g_{4}^{\left(2\right)}\right\}$, where the second group is always favored for the same reasons mentioned in Figure 4. Then, there is a second spontaneous differentiation in which the cell decides equally between the $g_{3}^{\left(2\right)}$ and $g_{4}^{\left(2\right)}$ cell types. When $h_{0}=0$, the colony can spontaneously differentiate into any of the four types of cells. Figure 5d illustrates a simulation in which the cell differentiates into $g_{1}^{\left(2\right)}$.

**Figure 6:**
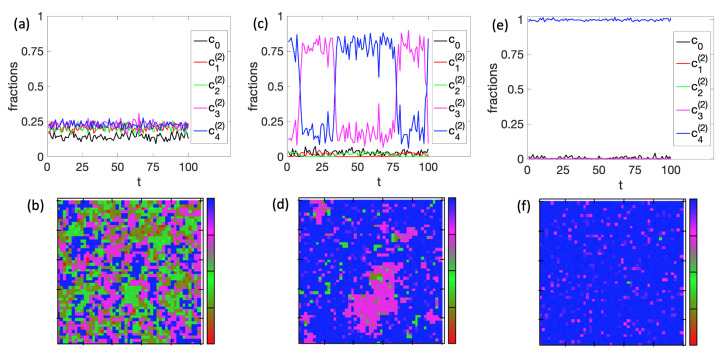
Cell-to-cell variability for r=2 with $h_{0}=0.5$ on a 50×50 tissue. First row ((**a**,**c**,**e**) shows time evolutions of the cell fractions, and the second row ((**b**,**d**,**f**) shows representative snapshots of the tissue states at time t=67 respectively. Temperature is T=3 for the first column ((**a**,**b**)), T=1.85 for the second column ((**c**,**d**)), and T=1 for the third column ((**e**,**f**)). The time unit is $10^{2}$ MC steps.

Next, we illustrate the cell-to-cell variability in the system. As an example, we used the parameters of Figure 5e and plotted the time evolution of the cell fractions, as well as representative snapshots at three different temperature points: T=3, T=1.85, and T=1 (Figure 6). At temperature T=3, precisely before the first differentiation, all types of cells co-exist throughout the simulation time (Figure 6a). The snapshot in Figure 6b illustrates this high level of cell variability. At T=1.85 (Figure 6c), where the second differentiation occurs, the system fluctuates significantly between $g_{3}^{\left(2\right)}$ and $g_{4}^{\left(2\right)}$. This fluctuation resembles the features of the second-order phase transition observed in 2D Ising models. In support of this assumption, the snapshot of Figure 6d shows the characteristic fractal-like structure of $g_{3}^{\left(2\right)}$ island sizes in a sea of $g_{4}^{\left(2\right)}$ cells. At low temperature T=1, the second differentiation has already taken place, and the decision to differentiate to cell $g_{4}^{\left(2\right)}$ was made for this particular simulation (Figure 6e,f). It must be underlined that, for this specific BN, the original cell $g_{0}$ can differentiate equally between the $g_{3}^{\left(2\right)}$ and $g_{4}^{\left(2\right)}$.

A second source of cell variability is due to the size of the colonies. For colonies of small size, there is always a measurable probability of having the first two types of cells, although the structure of the original cell does not favor this decision. This probability decreases significantly as the size of the tissue increases and almost vanishes for sizes greater than 20×20. This is due to weak ergodicity breaking that makes it difficult for the system to visit the first half of the state space. This theoretical interpretation may contribute to the cell-to-cell variability observed in small stem cell colonies.

Phase transitions can be studied in terms of the order parameters $c_{ww^{\prime }}=c_{w}-c_{w^{\prime }}$, where $w,w^{\prime }\in \left\{0,W_{r}\right\}$. Note that for each *r* there are M=m!/(m!(m−2)!), where $m=2^{r}+1$ unique fraction differences. Although this computation is manageable for r=1, it becomes increasingly difficult to solve for r≥2. In an ongoing project, we attempt to classify the phase transitions presented in Figure 4 and Figure 5 and compute the corresponding critical exponents. Preliminary results indicate that partial construction of the Waddington epigenetic landscape [43] is possible. Specifically, one can derive the quasi-potential $V\left(\left\{c_{ww^{\prime }}\right\}\right)\propto -\ln\left(P\left(\left\{c_{ww^{\prime }}\right\}\right)\right)$, where $P(\left\{c_{ww^{\prime }}\right\})$ is the joint probability distribution of all $\left\{c_{ww^{\prime }}\right\}$ [44,45]. Note that this scalar function is defined in an M-dimensional space. This construction can be simplified considerably in cases where some transitions are not possible. For example, in the model BN for r=2, the transitions from $g_{1}^{\left(1\right)}$ to $g_{3}^{\left(2\right)}$ and $g_{4}^{\left(2\right)}$ and from $g_{2}^{\left(1\right)}$ to $g_{3}^{\left(2\right)}$ and $g_{4}^{\left(2\right)}$ were not observable in the limited number of simulations performed for this section.

### 3.2. Self-Tuned Cell Differentiation

In Section 3.1, we showed that by manually tuning *T* and $h_{0}$ through the critical values, the system undergoes a series of symmetry-breaking events. In this section, we demonstrate that cells can collectively *self-tune* through a critical state that allows them to decide their fate. For simplicity, we set r=1 and neglect the local fields in Equation (5). This limit is equivalent to the standard Ising model. We recently showed that a mean-field approach of the Ising model with a negative feedback mechanism drives the system through a supercritical pitchfork bifurcation that can be interpreted as a cell fate decision [46]. Here, we apply this approach to the full Ising Hamiltonian Equation (5).

The heterogeneity of the system can be measured in terms of instantaneous “magnetization” $M_{t}=c_{2}^{\left(1\right)}\left(t\right)-c_{1}^{\left(1\right)}\left(t\right)$. A perfect mixture of $g_{1}^{\left(1\right)}$ and $g_{2}^{\left(1\right)}$ ($M_{t}=0$) corresponds to the pluripotent state of the cell [2]. On the contrary, $M_{t}=-1$ and $M_{t}=1$ correspond to homogeneous populations of cell types $g_{1}^{\left(1\right)}$ and $g_{2}^{\left(1\right)}$, respectively.

We consider an internal mechanism that allows the heterogeneity of a population, measured in terms of instantaneous magnetization of the tissue, $M_{t}$, to regulate the intrinsic noise of the population (temperature *T*):

(8) $$\begin{array}{c} \frac{dT}{dt}=\left|M_{t}\right|-\alpha T, \end{array}$$

where α is the relaxation coefficient. That is to say, Equation (8) captures the negative feedback response between cell–cell cooperativity and its intrinsic gene expression noise.

The model Hamiltonian for coupled Boolean networks (Equation (5)) with h=0 and $h_{0}=0$, combined with the internal temperature–magnetization feedback mechanism (Equation (8)), was simulated on tissues of size 32×32. The system was set to evolve for up to $t=8\times 10^{5}$ MC steps, where $N_{MC}=10$. Equation (8) was solved using the Euler method with a step size of $\Delta \tau =5\times 10^{-7}$, and the parameter α=0.8 was fixed. The model Boolean network from Figure 1 was instantiated for all cells with internal noise of q=0.02.

The simulation of the model begins with random gene states for all cells in the tissue, except for the CK node, which, without loss of generality, is set: $x_{c}=\left\{1\right\}$. We choose a high starting temperature of T=2.8 for the system, because it generates a natural state of hypothesized heterogeneity in pluripotent cells in the early stage of the embryonic stem cell cycle. Through negative feedback on instantaneous magnetization, Equation (8) then self-tunes the system towards the critical and then subcritical temperatures, where symmetry breaking triggers spontaneous differentiation.

**Figure 7:**
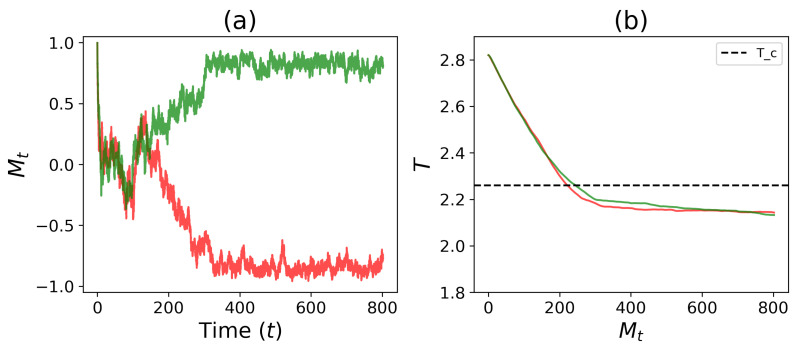
Simulations of a 32×32 Ising model with a self-tuning feedback equation (Equation (8)). Here, h=0, $h_{0}=0$, and α=0.8. (**a**) Magnetization trajectories show two systems are driven to $M_{t}\approx 0$ immediately upon initialization and eventually *self-tune* to two different homogeneous cell types ($M_{t}\approx -1$ or $M_{t}\approx 1$). (**b**) The simulations show that the temperature drop slows as it descends below the critical temperature ($T_{c}$) and eventually reaches a steady temperature. Combining (**a**) and (**b**), we see that as the feedback temperature reaches the Ising critical temperature ($T_{c}$), the magnetizations begin to diverge and tissue differentiates homogeneously to one of the two possible cell types. The time unit is $10^{2}$ MC steps with $N_{MC}=10$.

Figure 7a shows the time evolution of magnetization ($M_{t}$) for two independent simulations. Here, it can be seen that both simulations begin with $M_{t}=1$, which quickly approaches 0 over time. As time passes, the simulations decide and split in their magnetization paths ($M_{t}\approx 1$ and $M_{t}\approx -1$), resulting in differentiation of population cell types (which correspond to $g_{1}^{\left(1\right)}$ and $g_{2}^{\left(1\right)}$). Figure 7b shows the time evolution of the temperature trajectories of the two simulations. Here, the trajectories begin at T=2.8, and with time, the temperature drops below the critical temperature and eventually equilibrates to a subcritical temperature. Combined, we see that as the temperature reaches the Ising critical temperature ($T_{c}$), the tissue magnetizations diverge, with an equal chance of the system choosing one of the two cell types.

**Figure 8:**
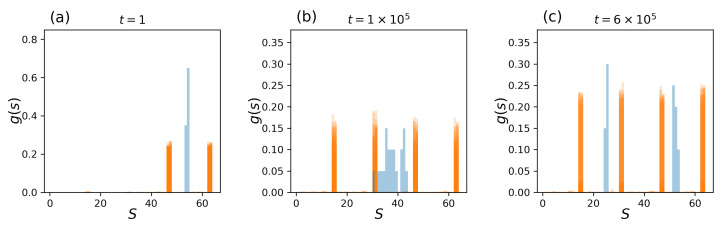
One hundred independent tissue simulations of a 32×32 Ising Hamiltonian (Equation (5) with the temperature–magnetization feedback mechanism (Equation (8)) are shown: (**a**) At time t=1, where the initial temperature is high (T=2.8), all cells are of cell type $g_{2}^{\left(1\right)}$. (**b**) At time $t=1\times 10^{5}$, where the tissues are $M_{t}\approx 0$, there is a mixture of $g_{1}^{\left(1\right)}$ and $g_{2}^{\left(1\right)}$ cell types from Figure 2 (left), and the average cell state at the colony-level remains unimodal, centered between the distributions $g_{1}^{\left(1\right)}\left(s\right)$ and $g_{2}^{\left(1\right)}\left(s\right)$. (**c**) At time $t=6\times 10^{5}$, the tissues decide on the fate of the cells with a drop in temperature to critical and subcritical points, and hence, $M_{t}\approx -1$ or $M_{t}\approx 1$. This results in a split, bimodal distributions of gene states at the cellular and colony level.

Observing tissue-level statistics provides additional insight into collective behaviors in pluripotent cells transitioning to two possible cell types. A total of 100 independent and identical tissue simulations of Figure 7 were carried out, and instantaneous state distributions G(t) and the average gene state (mean G(t) of a whole tissue) were collected at each time step. At the beginning of the simulation, t=1, the system was initialized with a high temperature point (T=2.8) and with the CK fixed to $x_{c}=\left\{1\right\}$, as in Figure 7. Trivially, all cell states are in $g_{2}^{\left(1\right)}$, and the average gene state, which describes the tissue-level distribution, is unimodal (Figure 8a). At time $t=1\times 10^{5}$, where the system reaches $M_{t}\approx 0$, two different cell types are probed, resulting in a mixture of $g_{1}^{\left(1\right)}$ and $g_{2}^{\left(1\right)}$ cell types, while the average state at the colony level remains unimodal, centered between $g_{1}^{\left(1\right)}$ and $g_{2}^{\left(1\right)}$ (Figure 8b). With time, the temperature self-tunes and reaches an equilibrium point below the critical temperature ($T_{c}$). At time $t=6\times 10^{5}$, approximately half of the tissues form a homogeneous cell type of $g_{1}^{\left(1\right)}$, and the other half form a cell type $g_{2}^{\left(1\right)}$. The tissue-level states reach a split bimodal distribution (Figure 8c). The system describes a full transition from a population of pluripotent tissue to two differentiated cell types. At the tissue level, this *unimodal–bimodal* transition at the critical junction of the phase transition occurs in several areas from mouse embryogenesis [12] to the development of cancer cell line [47].

As an application, *self-tuned differentiation* can replicate Okamoto et al.’s experimental work on the collective differentiation of mouse embryonic stem cells (mESCs) under strict conditions [12]. The authors have observed the gene expression levels of key transcription factors in mESC, Nanog, and Oct4 in the early stage of differentiation. According to the immunofluorescence markers of Venus and mKate2, which report Nanog and Oct4 gene expressions, respectively, colonies of mESCs exposed to leukemia inhibitory factor (LIF) demonstrated a high intensity of fluorescence, thus exhibiting single-state behavior in Nanog and Oct4. Here, LIF acts to enhance Nanog heterogeneity, in other words, to maintain the pluripotent state in the stem cell population. However, in the absence of LIF, high and low levels of Venus and mKate2 fluorescence were observed in cells, and cells are free to transition from pluripotent to differentiated state. This is termed *unimodal–bimodal transition* in the heterogeneity of gene expression levels at the cellular and colony level.

This cellular and colony-level unimodal–bimodal transition in the mESC distributions of a *pluripotent population* [6,48,49] is characterized by the multilayer Ising Hamiltonian (Equation (5)) with the temperature–magnetization feedback mechanism (Equation (8)) when pluripotent cells and differentiated cells are assumed to be *complementary* in gene states (i.e., they are induced by a fixed CK of r=1). Then, pluripotent and differentiated cell types form $g_{1}^{\left(1\right)}$ and $g_{2}^{\left(1\right)}$, which can exhibit the unimodal–bimodal transition with the self-tuning mechanism, as seen in Figure 8.

## 4. Conclusions

In this paper, we developed a model that describes the interplay between stem cell cooperativity and gene expression noise at the population level. Our model uses isogenic BNs to represent individual cell dynamics and a multilayer Ising Hamiltonian to describe the cell–cell interaction. This approach captures various cell signaling effects (paracrine and autocrine) and external gene expression interventions. The time evolution of this model is obtained by a hybrid MC method and synchronous update of BN states. We showed that a mixture of steady-state distributions can accurately represent the collective dynamics of coupled BNs. By interpreting each steady-state distribution as different types of cells, we characterized the compositions of stem cell populations with the aid of linear optimization techniques, standard spectral decomposition methods, and unsupervised machine learning algorithms.

Numerical analysis of our model in two dimensions revealed that by varying the system parameters through some critical values, the system undergoes a series of symmetry-breaking events that alter the dynamics of the original cell. Before these events, the system exhibits considerable cell-to-cell variability. This property resembles signatures of disorder–order phase transitions. We hypothesize that such transitions can be interpreted as the differentiation of the original cells into specialized cells. This interpretation supports the hypothesis that stem cells operate in a highly symmetric state (disorder state), and differentiated cells have a reduced symmetry state (order state). Furthermore, we showed that by introducing a differential equation that describes the negative feedback between cell cooperativity and intrinsic noise, the system can self-tune through the critical points and spontaneously differentiate into various types of cells. The number of different types of cells that each BN can generate strongly depends on the complexity of the cell–cell interaction and the structure of the original BN.

It is known that BNs can exhibit ordered, critical, and chaotic dynamics. How do the different BN dynamics affect the number and type of symmetry-breaking events observed in our model? Is criticality essential at the population level, locally at a single-cell level, or both? [36]). In this work, we showed that our model generates a number of different cell states. Depending on the structure of the BN, some states are stable and others are metastable. Preliminary results show that metastable cell states can emerge in certain conditions in cell tissues. What is the biological interpretation of such metastabilities? Here, we only considered ferromagnetic cell–cell interaction (i.e., J>0). Can a different type of interaction (e.g., antiferromagnetic or random field) lead to other types of phase transition? Is a spin-glass phase transition possible in our model, and how could this affect collective cell dynamics? These questions, along with the partial construction of the epigenetic landscape and characterization of the phase transitions, will be studied in detail in a future publication [46].

## Data Availability

Not Applicable.

## Appendix A

**Table A1:** Table of parameters, definitions, and parameter values utilized in the model development and simulations. There are two control parameters: the intercellular intrinsic noise (*T*) and the tendency of the system to retain its original BN dynamics ($h_{0}$). Both parameters are measured in *J* units.

Parameter Notation	Definition	Parameter Values
*k*	BN connectivity	2
*p*	Boolean function bias	0.5
*q*	perturbation probability	0.1,0.02
*r*	control kernel size	1,2
*J*	interaction strength/energy unit	1
*h*	external field constant	0
α	relaxation coefficient	0.8
T	simulation time (MC steps)	$10^{4}$
$t_{eq}$	equilibration time (MC steps)	$10^{4}$
